# Newly synthesized chitosan-nanoparticles attenuate carbendazim hepatorenal toxicity in rats via activation of Nrf2/HO1 signalling pathway

**DOI:** 10.1038/s41598-022-13960-1

**Published:** 2022-06-15

**Authors:** Elshazly Mo, Yasmin A. Ebedy, Marwa A. Ibrahim, Khaled Y. Farroh, Eman I. Hassanen

**Affiliations:** 1 Pathology Department, Faculty of Veterinary Medicine, Cairo University, P.O. Box 12211, Giza, Egypt; 2 Biochemistry Department, Faculty of Veterinary Medicine, Cairo University, Giza, Egypt; 3 Nanotechnology and Advanced Materials Central Lab, Agricultural Research Center, Giza, Egypt

**Keywords:** Biochemistry, Biomarkers, Gastroenterology, Nephrology, Pathogenesis

## Abstract

Widespread application of carbendazim (CBZ) is a major environmental impact because of its residues that caused multi-organ dysfunction. Recently, Chitosan nanoparticles (CS-NPs) are extensively used as nanocarriers due to their non-toxic and biodegradable nature. Therefore, the current study aimed to investigate the possible mechanistic pathway of modified CS-NPs to reduce the hepatic and nephrotoxicity of CBZ in rats. CS-NPs were synthesized by the ionic gelation method by using ascorbic acid instead of acetic acid to increase its antioxidant efficiency. Twenty-adult male Wistar rats were grouped (n = 5) as follows: Group (1) negative control, group (2) received CS-NPs, group (3) received CBZ, and group (4) co-administered CS-NPs with CBZ. Rats received the aforementioned materials daily by oral gavage for 28 days and weighed weekly. The results revealed that CBZ receiving group showed severe histopathological alterations in the liver and kidney sections including cellular necrosis and interstitial inflammation confirmed by immunostaining and showed marked immunopositivity of iNOS and caspase-3 protein. There were marked elevations in the serum levels of ALT, AST, urea, and creatinine with a significant increase in MDA levels and decrease in TAC levels. Upregulation of the Keap1 gene and down-regulation of Nrf2 and HO-1 genes were also observed. Co-treatment of rats by CS-NPs with CBZ markedly improved all the above-mentioned toxicological parameters and return liver and kidney tissues to normal histological architecture. We concluded that CBZ caused hepatorenal toxicity via oxidative stress and the Nrf2/HO-1 pathway and CS-NPs could reduce CBZ toxicity via their antioxidant, anti-apoptotic, and anti-inflammatory effects.

## Introduction

Carbendazim (CBZ, methyl N-(1H-benzimidazol-2-yl) carbamate) is a systemic fungicide that is commonly used in agriculture to combat fungal infections^1^. CBZ is extensively used as a biocide for the protection of product categories such as film, leather, rubber, fiber, polymerized materials, and building facades, according to European Union (EU) regulation number 528/2012^2^. CBZ is a huge global concern because it is frequently transferred via rains to areas where it poses harm to the environment, animal health, and human health^3^. The residual concentration of CBZ in farmland is about 0.18–1.74 mg/kg soil, while those in some fruits and vegetables were 0.37 and 0.60 mg/kg^4^. Moreover, CBZ is considered as a persistent environmental contaminant because its benzimidazolic ring which is difficult to break so, its degradation is slow^5^. Several countries have reported that CBZ is frequently detected in soil and water for 3 days to 12 months^6,7^. Humans are exposed to carbendazim by direct (inhalation and cutaneous contact) or indirect (ingestion of polluted water, food, and occupational exposure) pathways^8^. CBZ gets metabolized to 5-hydroxy-2-benzimidazole carbamate and 5,6-HOBC-N-oxides (5,6- HOBC-N-oxides) that are poorly catabolized and retained in tissues such as gonads, liver, adrenals, adipose, skin and other organs. The CBZ and its retained metabolites in tissues have been shown to cause infertility and destruction of testicles in rats and mice^9,10^. It was discovered to induce a variety of negative health effects in animal models, including hepatotoxicity, reproductive toxicity, and endocrine disruption^11^. Overproduction of reactive oxygen species (ROS), which interact with various cellular constituents such as DNA, proteins, and lipids, causes cell and DNA damage, which is a primary factor in the toxicity generated by carbendazim^12,13^.

Nanotechnology is the study of extremely small structures with dimensions ranging from 0.1 to 100 nm^14^. Nanoparticles (NPs) have significantly increased their use due to their reduced size and unique features^15^. They're used in a variety of biological, biotechnological, and optical applications^16^. Chitosan is a type of natural polysaccharide polymer made from chitin^17^. It is a non-toxic, biodegradable, and biocompatible agent that has been studied extensively in a variety of biomedical studies, including vaccine delivery, preparation of hydrogels, films, and natural fiber^18,19^. Recently, chitosan nanoparticles (CS-NPs) are extensively used as nanocarriers for various biological agents such as drugs, proteins, and genes^20^. CS-NPs are also used in the agricultural field as plant growth promoters and protectors and also used as encapsulated agents for several agrochemicals^21^. It is reported that the encapsulation of pesticides with non-toxic NPs as CS-NPs can control the release of pesticides^22^.

The adverse health effects of CBZ residues on non-target organisms have directed the science to search for methods to minimize these effects. CS-NPs are known to have many benefits in medicine and agriculture due to their antimicrobial and chelating properties in addition to their biocompatible nature, but their pathophysiology in the body hasn’t been fully elucidated. The in-vivo antioxidant effects of CS-NPs and their mechanisms didn’t report until now. Therefore, the current study aimed to investigate the ameliorative effect of CS-NPs against CBZ-induced hepatorenal oxidative stress damage and discuss the role of Keap1/Nrf2/HO-1 signaling pathway.

## Materials and methods

### Chemicals

Carbendazim (purity: 97%, CAS number: 10605–21-7, MW: 191.19 kg/Mol), Chitosan (molecular weight 50,000–190,000 Da, degree of deacetylation 75–85%, viscosity 20–300 cP), ascorbic acid and other chemicals used for nanoparticles preparation were purchased from Sigma-Aldrich Chemicals Co., St. Louis, MO, USA.

### Chitosan nanoparticles preparation and characterization

Chitosan nanoparticles were synthesized by the ionic gelation method as described by Agarwal et al.^23^ but, with some modification. Briefly, CS aqueous solution (0.1% W/V) was prepared by dissolving 0.1 g chitosan in ascorbic acid solution (0.4% W/V) with stirring for 1 h. Afterward, 0.033 g TPP was dissolved in 10 ml deionized water and added dropwise to the chitosan solution with continuous stirring. The resulting chitosan particle suspension was centrifuged at 12000 g for 30 min, and the pellet was resuspended in deionized water. The chitosan nanoparticles suspension was then freeze-dried before further use or analysis.

The elemental and physicochemical structures of the prepared CS-NPs were characterized using FTIR (Thermo Fisher Scientific Inc., Pittsburgh, PA, USA) and XRD (X’Pert Pro, Analytical, Netherlands). The wavelength was measured using UV-spectrophotometer (Cary 5000, Varian, Australia). The average size and zeta potential were determined by photon correlation spectroscopy and laser doppler anemometry, respectively, in triplicate using Nano-Zetasizer 3,000 HS (Malvern Instruments, Malvern, UK). The morphological shape of CS-NPs was determined by using transmission electron microscopy (TEM, Tecnai G20, FEI, Netherlands). Additionally, periodical DLS analysis were done to ensure the stability of NPs in the solution to avoid the particles aggregation.

### Animals and experimental design

All the procedures of the experimental study were designed in accordance with the ARRIVE guidelines (PLoS Bio 8(6), e1000412,2010​) and approved by the institutional animal care and use committee (IACUC) of Cairo University (protocol number: Vet CU12102021369), Cairo, Egypt. Twenty mature male albino Wistar rats weighing 170–200 g were purchased from VACSERA, Helwan, Egypt. All rats were caged in plastic cages (5 rats/cage) in a well-ventilated environment and acquired 12 h of light every day. They had been consumed dry commercial standard pellets and had access to tap water ad libitum through the trial time frame. Two weeks of acclimatization were done before the start point of the experiment to ensure their health status.

Rats were randomly allocated into 4 groups (n = 5) using the block randomization strategy and received the treatments every day by means of oral gavage for 28 days. Group (1) received normal saline and was kept as a control negative. Group (2) received 5 mg/kg bwt CS-NPs. Group (3) received 300 mg/kg bwt CBZ corresponding to 1/20 LD50 and equivalent to human dose of 42.8 mg/kg bwt^24,25^. Group (4) received CS-NPs + CBZ at the same doses mentioned previously. The dose of CS-NPs was selected in accordance with the previous study^26^. CS-NPs were freshly prepared once a week to avoid particles aggregation. All rats were observed daily for any signs of illness and weighted weekly throughout the experimental period.

### Sampling

After 28 days postdosing, all rats were anesthetized with Ketamine and Xylazine then blood samples were collected from the tail vein and centrifuged at 3500 rpm for 15 min to obtain serum samples preserved at -20ºC for further use. Afterward, all rats were euthanized by cervical dislocation to collect liver and kidney tissue specimens that were divided into two parts. The first part was fixed in 10% neutral buffered formalin to carry out the histopathological and immunohistochemical assessment, while the second part was preserved at -80ºC till used for oxidative stress evaluations and molecular studies.

### Liver and kidney biomarkers

Serum levels of alanine aminotransferase (ALT), aspartate aminotransferase (AST), creatinine, and blood urea nitrogen (BUN) were estimated following the directions of the manufacturer’s kits (Bio-diagnostic Co., Egypt).

### Oxidative stress evaluation

Malondialdehyde (MDA) level, reduced glutathione (GSH) content, catalase activity, and total antioxidant capacity (TAC) were measured in hepatorenal tissue homogenates and serum samples following the manufacturer kits instruction (Bio-diagnostic Co., Egypt).

### Histopathological examination

Formalin‐fixed liver and kidney tissue specimens were processed by using an ascending grade of alcohol and xylene. Then, the specimens were embedded in paraffin wax, cut by the ordinary microtome at 4.5 μm, stained with H&E, and then examined under light Olympus BX43 microscope and the images were captured by Olympus DP27 camera linked to CellSens dimensions software (Product Version, 1.13; Core Version, XV 3.12 (Build 13,479)) https://www.olympus-lifescience.com/en/software/cellsens/^27^.

The semiquantitative multiparametric classical scoring system was used to assess the degree of severity of the pathological changes in liver and kidney sections in accordance with the protocol mentioned by Khalaf et al.^28^. The diffuse microscopic lesions were blindly evaluated and scored as slight, mild, moderate, and severe, on five-pointed ordinal scale, as the accompanying (0 = normal histology, 1 < 25%, 2 = 25:50%, 3 = 50:75%, and 4 > 75% tissue damage). While grading scheme for focal lesions were blindly assessed as follows: (0) no foci; (1) < 3 foci; (2) 3–6 foci; (3) 7–12 foci; (4) > 12 foci/ field at low power (100X)^29^.

### Immunohistochemical staining

Localization of Caspase-3 and inducible nitric oxide synthase (iNOS) were carried out on formalin‐fixed paraffin‐embedded liver and kidney sections. Briefly, the slides were incubated with different primary antibodies (Abcam, Ltd.) at 1/200 dilutions, then incubated with Peroxidase Block (Sakura BIO) and the reagent needed for the identification of the antigen‐antibody complex (Power‐Stain 1.0 Poly HRP DAP Kit; Sakura). The sections were treated with 3, 3′‐diaminobenzidine chromogen substrate for 10 min and counterstained by Hematoxylin and inspected by light Olympus BX43 microscope and caught by Olympus DP27 camera connected to CellSens dimensions software (Olympus).

Absolute quantitative scoring of the immunostaining reactions was done by using ImageJ software. The average size of various immunostaining reactivity was determined as the percentage of specific positive area concerning the total area per low power magnification (100X).

### Quantitative RT-PCR for Nrf2, HO-1, and Keap1 genes

The Total RNA was extracted from both the liver and kidney tissues using EasyRNA™ Cell/Tissue RNA Mini Kit (Biovision #K1337) according to the manufacturer’s instructions. The isolated RNAs were used for the synthesis of first-strand cDNA using Super Script Reverse Transcriptase (Thermoscientific) according to the manufacturer’s instructions. Quantitative real-time PCR was performed using Power Track™ SYBR Green Master Mix Applied Biosystems™ on an ABI Prism Step One Plus Real-Time PCR System (Applied Biosystems) according to the manufacturer’s instructions. The primer sets of the tested genes were gathered in Table 1 and the target mRNA expression was normalized to ACTB.

**Table 1:** The primer set for the measured genes.

	Sense	Antisense	Amplicon	Accession no
*Nrf-2*	TGTAGATGACCATGAGTCGC	TCCTGCCAAACTTGCTCCAT	159	NM_031789.2
*Ho-1*	AGCGAAACAAGCAGAACCCA	ACCTCGTGGAGACGCTTTAC	166	*NM_012580.2*
*Keap-1*	ATGTGATGAACGGGGCAGTC	AAGAACTCCTCCTCCCCGAA	190	*NM_057152.2*
*ACTB*	CCGCGAGTACAACCTTCTTG	CAGTTGGTGACAATGCCGTG	297	NM_031144.3

*Nrf-2* nuclear factor-erythroid 2-related factor 2, *Ho-1* heme oxygenase-1, *Keap-1* kelch like ECH associated protein 1, *ACTB* ACTB actin beta gene.

### Statistical analysis

The obtained data were expressed as means ± error of mean (SEM) and analyzed by using SPSS version 24.0 software (SPSS Inc., Chicago, IL, USA). One-way analysis of variance (ANOVA) followed by Tukey Post Hoc-test Comparison of means was performed. A value of *P* ≤ 0.05 was considered statistically significant. Nonparametric values as microscopic lesion scoring values were represented as median values and analyzed using Kruskal Wallis *H*-test followed by Mann–Whitney *U-*test.

### Ethics approval and consent to participate

All Institutional and National Guidelines for the care and use of animals were followed.

## Results

### Characterization of CS-NPs

**Figure 1:**
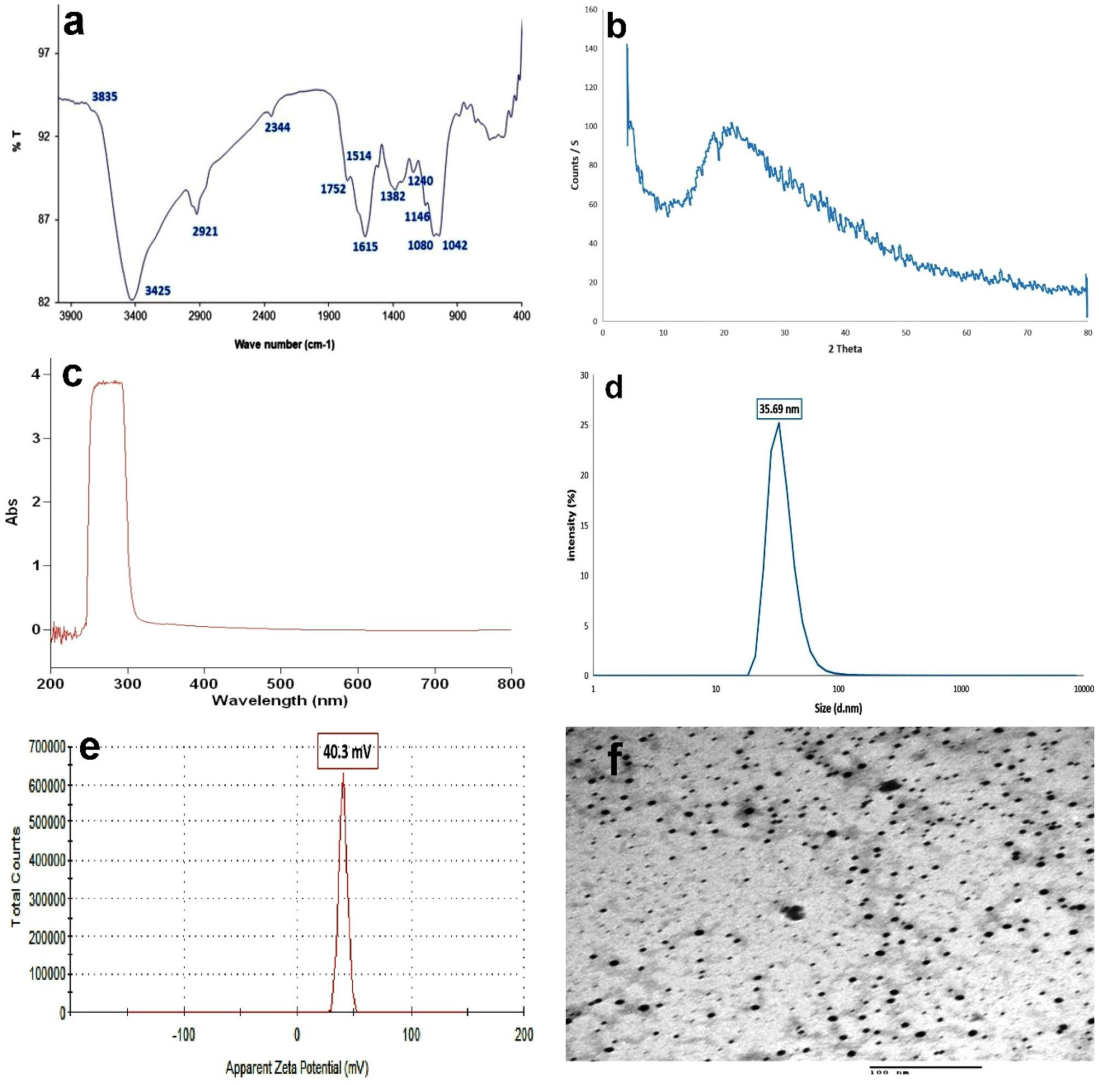
Characterization of the prepared CS-NPs. (**a**) Elemental structure by FTIR. (**b**) Physicochemical structure by XRD. (**c**) Peak absorption. (**d**) The particle size distribution curve. (**e**) Average zeta potential. (**f**) Transmission electron microscope photograph.

The results of FTIR showed bands at 3425 and 3835/cm representing OH groups. The band at 2921/cm represents the N–H and C-H bonds in CH2 and CH3 groups respectively, while the C=O bonds are located at positions 2344 and 1752/cm. The bands at 1615 and 1514/cm represent the C=C bonds. The methyl group is located at 1382/cm position. The band at 1240/cm is due to the C-O group. The band at 1146 /cm is for the antisymmetric stretching of C–O–C bridge. Peaks at 1080 and 1042/cm correspond to C-O bonds. The bands below 1000 may be due to the CH-CH2 and CH=CH groups with its cis and transpositions (Fig. 1a). XRD physicochemical characterization showed characteristic peaks of 2θ around 10° to 35° (Fig. 1b). In addition, UV-spectrophotometer revealed particles with broad peak absorption (3.8%) at wavelength 256–292 nm (Fig. 1c). Moreover, the dynamic light scattering (DLS) showed a particle size distribution curve (22.3%) at 35 nm and a zeta potential peak (100%) at 40.3 mV **(**Figs. 1d,e). TEM photograph showed spherical to amorphous shaped particles of CS-NPs (Fig. 1f).

### Clinical signs and body weights

There were no clinical signs observed on rats in any groups throughout the experimental period. The average body weights (ABW) were almost stable among all groups during the first three weeks of the experiment. At 4th week, the ABW significantly decrease in CBZ receiving group compared with the control group but, was quite similar in other groups (Table 2).

**Table 2:** The effect of carbendazim (CBZ) and/or chitosan nanoparticles (CS-NPs) on the average body weights.

	Control	CS-NPs	CBZ	CBZ + CS-NPs
W 1	196.33 ± 12.23^a^	193.66 ± 8.17^a^	200 ± 7.50^a^	200 ± 5.22^a^
W 2	215.66 ± 9.38^a^	218.33 ± 11.56^a^	216.8 ± 8.01^a^	214.4 ± 7.52^a^
W 3	220.66 ± 10.68^a^	214.66 ± 9.70^a^	216.4 ± 7.56^a^	222.6 ± 6.86^a^
W 4	220.33 ± 9.70^a^	218.33 ± 9.52^a^	202.2 ± 7.57^b^	222.4 ± 8.53^a^

All data are presented as mean ± SEM (n = 5 rats/ group). Values bearing different superscripts (a, b) at the same row means significant at *P* ≤ 0.05.

### Biochemical parameters

A significant increase in the serum levels of ALT, AST, BUN and creatinine were observed in CBZ receiving group compared with other groups. On the other hand, there was a significant decrease in the aforementioned biomarkers in the group receiving CS-NPs + CBZ compared with CBZ group but still high when compared with the control group (Table 3).

**Table 3:** The effect of carbendazim (CBZ) and/or chitosan nanoparticles (CS-NPs) on some liver and kidney biomarkers.

Group/parameter	Control	CS-NPs	CBZ	CBZ + CS-NPs
ALT (U/L)	23.67 ± 1.11^a^	22.64 ± 1.52^a^	64.55 ± 3.24^b^	37.15 ± 3.16^c^
AST (U/L)	52.64 ± 3.14^a^	51.61 ± 3.22^a^	61.51 ± 3.51^b^	55.29 ± 2.81^ac^
Urea (mg/dl)	32.52 ± 1.54^a^	31.33 ± 1.30^a^	65.15 ± 2.37^b^	40.58 ± 2.24^c^
Creatinine (mg/dl)	0.64 ± 0.08^a^	0.61 ± 0.09^a^	1.32 ± 0.07^b^	0.91 ± 0.02^c^

All data are presented as mean ± SEM (n = 5 rats/ group). Values bearing different superscripts (a, b, c) at the same row means significant at *P* ≤ 0.05.

### Oxidative stress evaluations

There was a significant increase in MDA levels in both liver and kidney homogenates obtained from CBZ receiving group compared with other groups. On the other hand, the MDA levels were significantly decreased in the group receiving CS-NPs + CBZ compared with CBZ receiving group. Regarding TAC content, the lowest value was noticed in the group receiving CBZ while other TAC values were quite close to each other. While the levels of GSH and catalase activity were markedly decreased in CBZ receiving group compared with other groups (Table 4).

**Table 4:** The effect of carbendazim (CBZ) and/or chitosan nanoparticles (CS-NPs) on some oxidative stress markers.

Group/parameter	Control	CS-NPs	CBZ	CBZ + CS-NPs
**Liver**				
MDA (nmol/g)	21.13 ± 0.72^a^	20.98 ± 3.05	45.61 ± 1.27^b^	25.82 ± 2.90^c^
GSH (mg/g)	13.2 ± 0.15^a^	15.1 ± 2.52^ab^	9.3 ± 2.67^c^	12.1 ± 5.12^a^
Catalase (U/g)	65 ± 0.56^a^	75.6 ± 5.18^b^	46.7 ± 3.53^c^	59 ± 2.23^ad^
**Kidney**				
MDA (nmol/g)	30.38 ± 1.62^a^	27.57 ± 5.41^ac^	74.39 ± 2.81^b^	15.82 ± 1.36^c^
GSH (mg/g)	43 ± 2.15^a^	41.2 ± 0.65^a^	16.5 ± 2.02^b^	45.2 ± 1.56^ac^
Catalase (U/g)	35 ± 1.72^a^	36.5 ± 0.16^a^	15.9 ± 5.19^b^	29 ± 1.81^c^
**Serum**				
TAC (nmol/L)	1.91 ± 0.32^a^	2.92 ± 0.15^b^	0.53 ± 0.02^c^	1.14 ± 1.08^d^

All data are presented as mean ± SEM (n = 5 rats/ group). Values bearing different superscripts (a, b, c) at the same row means significant at P ≤ 0.05.

### Histopathological examination

**Figure 2:**
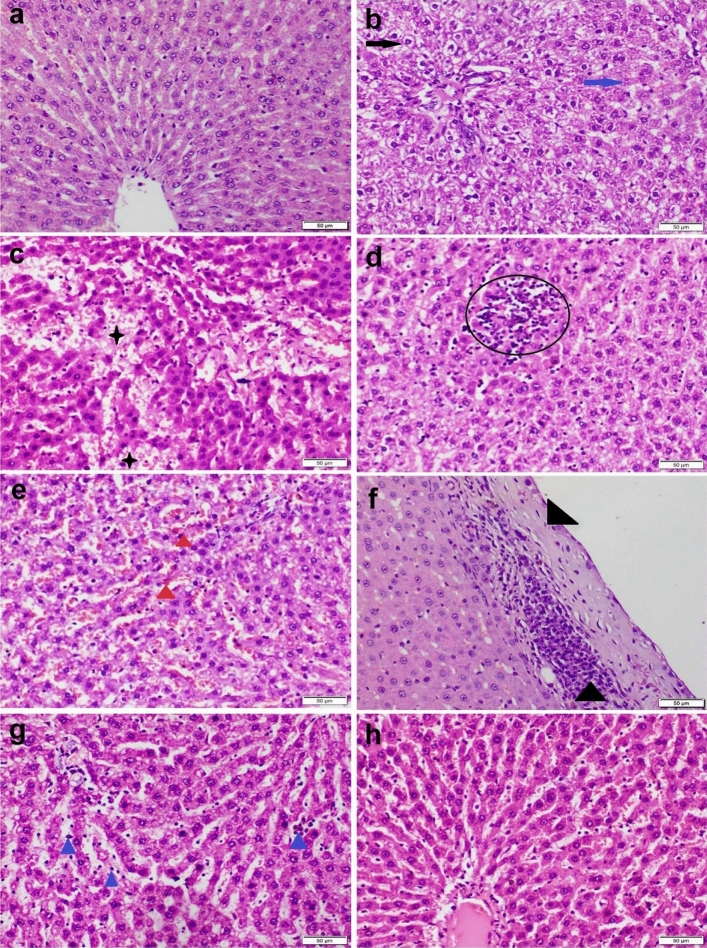
Photomicrograph of liver tissue sections stained by H&E representing; (**a**) Control group showing normal histological structure. (**b**–**f**) CBZ receiving group showing diffuse hepatocellular cytoplasmic vacuolization (black arrows) and sparse cell necrosis (blue arrows), hemorrhage (black stars), focal necrotic area with inflammatory cells infiltration (circle), sinusoidal congestion (red triangle), thickening of the Glissonian capsule with extensive inflammatory cells infiltration (black triangles). (**g**–**h**) CS-NPs + CBZ receiving group showing; (**g**) Sinusoidal lymphocytic exocytosis (blue triangle). (**h**) Normal histological structure.

Liver tissue sections obtained from the control group and those receiving CS-NPs showed ordinal histological architecture (Fig. 2a). On the other side, liver sections obtained from CBZ receiving group showed severe histopathological alterations. Severe diffuse hepatocellular vacuolar degenerations with sparse cell necrosis were observed in most sections (Fig. 2b). There were multifocal to convalescent areas of hemorrhage within the hepatic parenchyma (Fig. 2c). Vascular congestion and sinusoidal exocytosis (Fig. 2d) were the constant lesions noticed in all sections. Portal triad showed congestion, fibroplasia and mononuclear inflammatory cells infiltration (Fig. 2e). Most sections showed thickening in the Glissonian capsule by fibrosis and inflammatory cells infiltration (Fig. 2f). Co-administration of CS-NPs with CBZ markedly improved the microscopic picture of liver tissue (Fig. 2g). Neither hepatocellular cytoplasmic vacuolization nor portal inflammation was recorded. Mild to moderate vascular congestion and sinusoidal lymphocytosis were observed (Fig. 2h).

**Figure 3:**
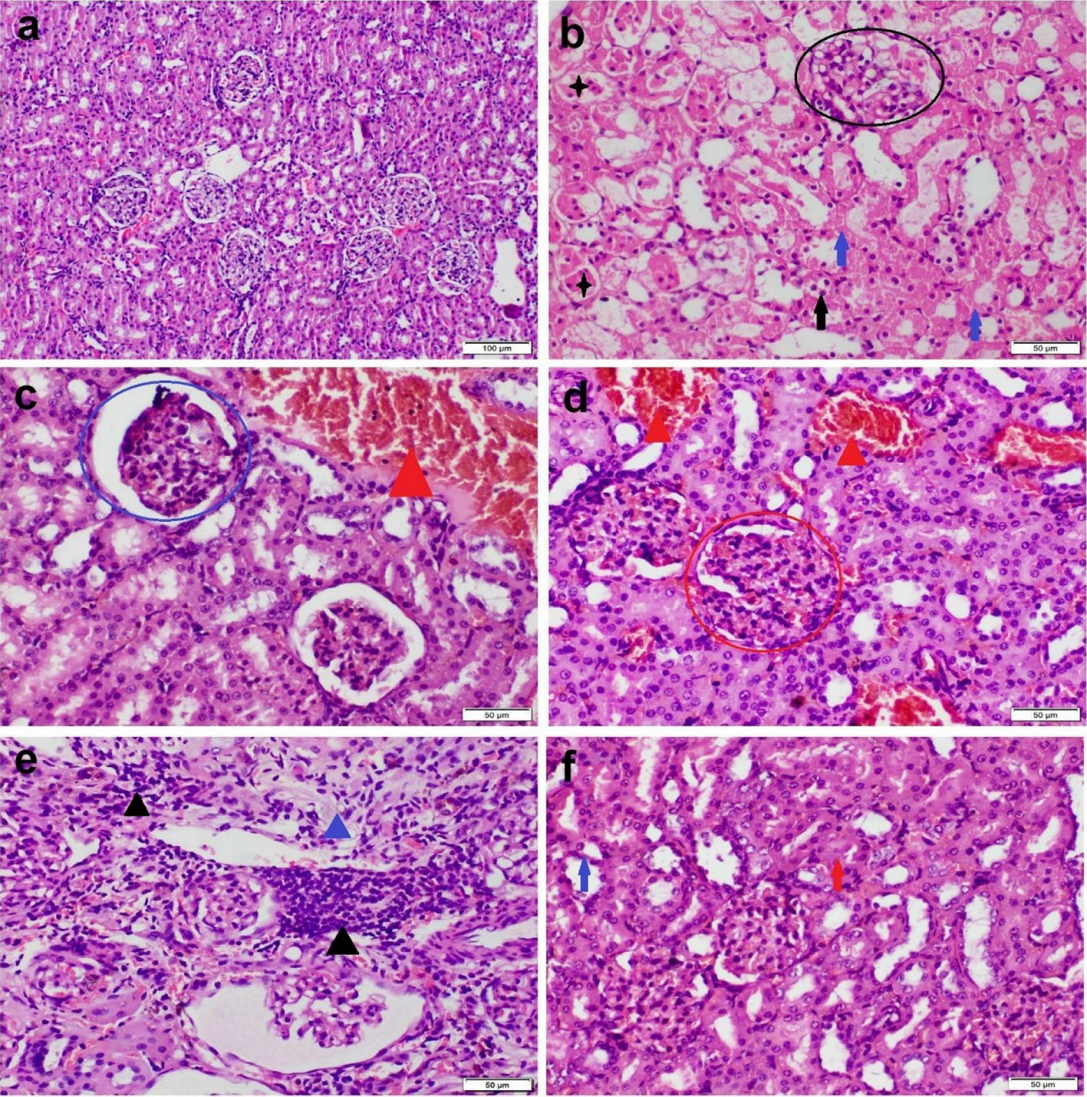
Photomicrograph of kidney tissue sections stained by H&E representing; (**a**) Control group with normal histological structure. (**b**–**e**) CBZ receiving group showing severe cytoplasmic vacuolization (black arrows) and necrosis (blue arrows) of the tubular epithelium, intraluminal cellular cast (black stars), glomerular degeneration (black circle), widening of the bowmen’s space with atrophy of the glomerular tuft (blue circle), glomerular congestion (red circle), interstitial vascular congestion (red triangle), inflammatory cells infiltration (black triangle), and fibroplasia (blue triangle). (**f**) CS-NPs + CBZ receiving group showing cellular swelling (red arrow) and necrosis (blue arrow) in some tubular epithelium.

Kidney tissue sections obtained from the control group and those receiving CS-NPs showed normal histological structure (Fig. 3a). On the other side, kidney tissue sections obtained from CBZ receiving group showed severe histopathological alterations. The most prominent lesions observed were several degenerative changes and necrosis of the tubular epithelium (Figs. 3b,c). Most of the glomeruli showed atrophy of the capillary tuft and widening of the bowman’s space (Fig. 3d). There were vascular congestion, hemorrhage, and inflammatory cells infiltration in the interstitial tissue (Fig. 3e). Co-administration of CS-NPs with CBZ markedly improved the microscopic picture of kidney sections. Neither tubular degenerations nor interstitial inflammations were recorded. Mild to moderate interstitial and glomerular congestion were recorded (Fig. 3f).

The results of the microscopic lesion scoring revealed a significant increase in all the examined histopathological parameters of both liver and kidney sections obtained from CBZ receiving group compared with other groups. Otherwise, group co-administered CS-NPs with CBZ showed a significant reduction in all lesions scoring of both liver and kidneys compared with CBZ receiving group and nearly similar to those of the control group in some parameters (Table 5).

**Table 5:** The hepatic and renal microscopic lesion scoring in different treatment groups.

	Control	CS-NPs	CBZ	CBZ + CS-NPs
**Hepatic microscopic lesion scoring**				
HCD	0 ^a^	0 ^a^	4 ^b^	2 ^c^
HCN	0 ^a^	0 ^a^	4 ^b^	1 ^c^
Inflammation	0 ^a^	0 ^a^	4 ^b^	1 ^c^
Portal fibrosis	0 ^a^	0 ^a^	3 ^b^	0 ^a^
Perihepatitis	0 ^a^	0 ^a^	3 ^b^	0 ^a^
**Renal microscopic lesion scoring**				
RTD	0 ^a^	0 ^a^	4 ^b^	2 ^c^
RTN	0 ^a^	0 ^a^	4 ^b^	1 ^c^
Hemorrhage	0 ^a^	0 ^a^	2 ^b^	0 ^a^
Inflammation	0 ^a^	0 ^a^	4 ^b^	0 ^a^
Glomerular atrophy	0 ^a^	0 ^a^	4 ^b^	0 ^a^

Values represented as median (n = 7 microscopic field in 5 sections representing 5 rats/group). Different letter at the same row means significant difference at *P* < 0.05.

(0) normal histology, (1) mild changes < 25% tissue damage, (2) moderate changes 25–50% tissue damage, (3) severe changes 50–75% tissue damage, (4) extremely severe changes > 75% tissue damage.

*HCD* hepatocellular degeneration, *HCN* hepatocellular necrosis, *RTD* renal tubular epithelial degeneration, *RTN* renal tubular epithelial necrosis.

**Table 6:** Mean percentage area of different immune markers expression in liver and kidneys.

	Control	CS-NPs	CBZ	CBZ + CS-NPs
**Immune expression in liver tissue**				
Casp-3 (%)	0.5 ± 0.1^a^	0.3 ± 0.02^a^	13 ± 3.5^b^	5 ± 0.8^c^
iNOS (%)	0 ± 0^a^	0 ± 0^a^	21 ± 4.6^b^	7 ± 1.6^c^
**Immune expression in kidney tissue**				
Casp-3 (%)	0.5 ± 0^a^	0.5 ± 0.2^a^	15 ± 1.7^b^	3 ± 0.8^c^
iNOS (%)	0.1 ± 0^a^	0 ± 0^a^	18 ± 2.1^b^	6 ± 2.4^c^

Values represented as Mean ± SEM (n = 7 microscopic field in 5 sections representing 5 rats/group). Values with different letters at the same row means significant difference at *P* ≤ 0.05.

### Immunohistochemical staining

**Figure 4:**
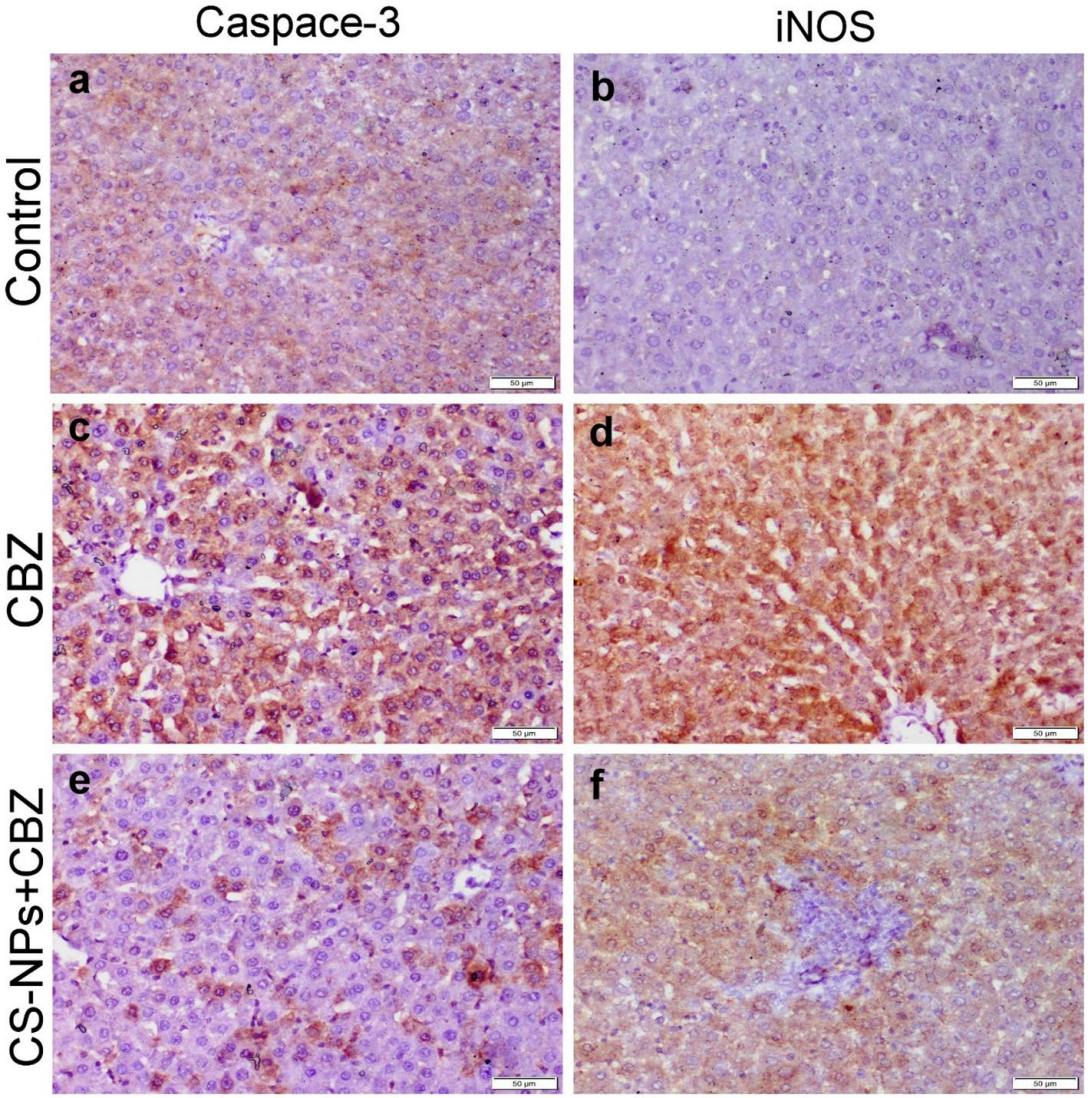
Photomicrograph of liver tissue sections representing the localization of casp-3 and iNOS protein in different treated groups. (**a**, **b**) Control negative group showing normal slight to negative casp-3 and iNOS protein expression. (**c**, **d**) CBZ receiving group showing strong positive casp-3 and iNOS protein expression in the tubular epithelium and interstitial inflammatory cells. (**e**, **f**) CS-NPs + CBZ receiving group showing weak expressions of both casp-3 and iNOS proteins.

**Figure 5:**
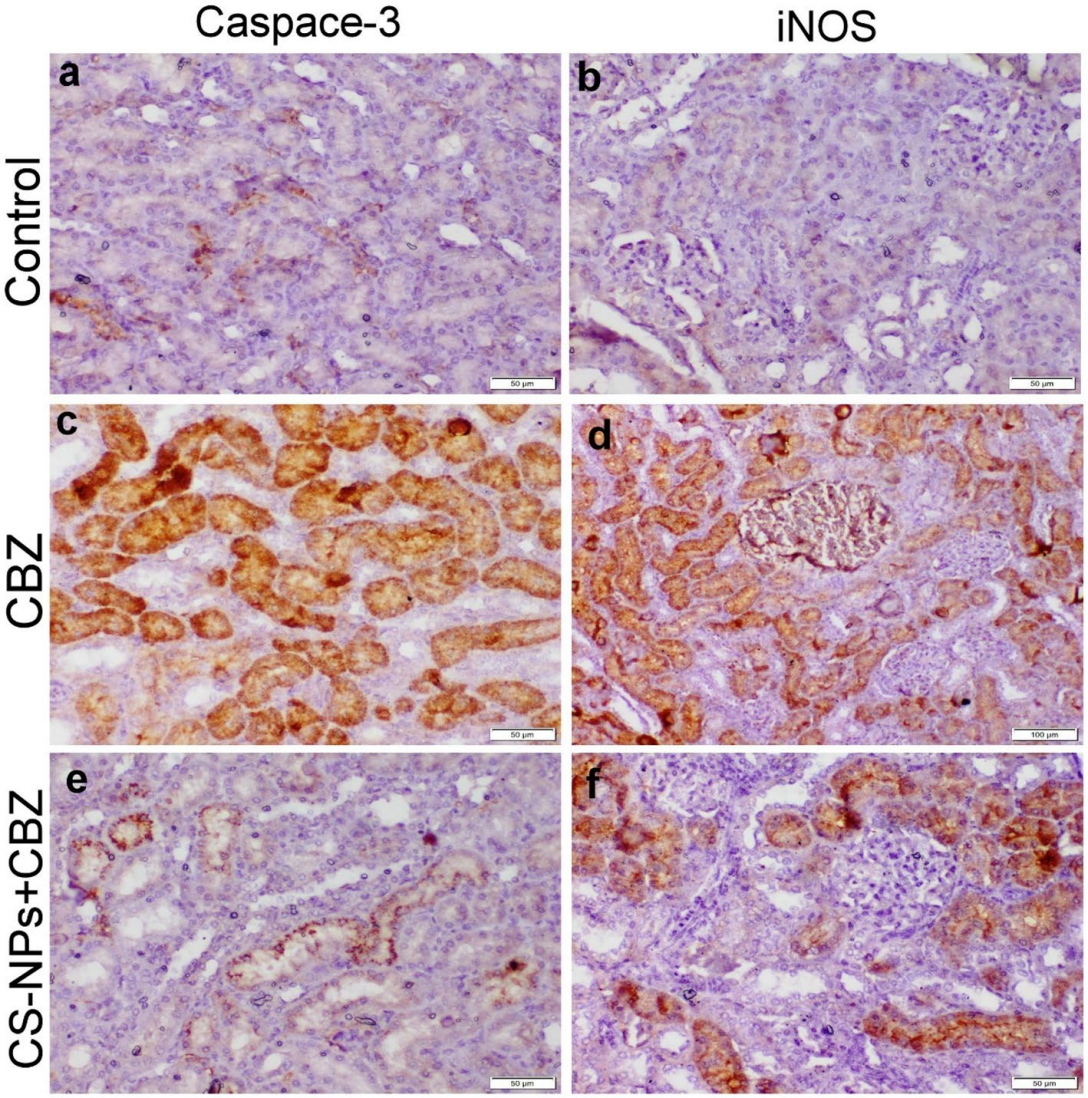
Photomicrograph of kidney tissue sections representing the localization of casp-3 and iNOS protein in different treated groups. (**a**, **b**) Control negative group showing normal slight to negative casp-3 and iNOS protein expression. (**c**, **d**) CBZ receiving group showing strong positive casp-3 and iNOS protein expression in the tubular epithelium and interstitial inflammatory cells. (**e**, **f**) CS-NPs + CBZ receiving group showing weak expressions of both casp-3 and iNOS proteins.

Both liver and kidney sections obtained from the control group and those receiving CS-NPs showed normal negative to slight positive expression of casp-3 and iNOS. On the other hand, liver and kidney sections of CBZ receiving group showed strong immunopositivity of casp-3 and iNOS. Co-administration of CS-NPs with CBZ had the ability to reduce all the above-mentioned immune markers (Figs. 4 and 5). The results of the quantitative scoring reported in Table 6 showed the highest immunopositive percentage area for casp-3 and iNOS in liver and kidney sections of CBZ group compared with other groups. Otherwise, the positive percentage area of both immune markers was significantly decreased in the liver and kidney sections of CS-NPs cotreatment group compared with CBZ group.

### Quantitative RT-PCR for Nrf2, HO-1, and Keap1 genes

**Figure 6:**
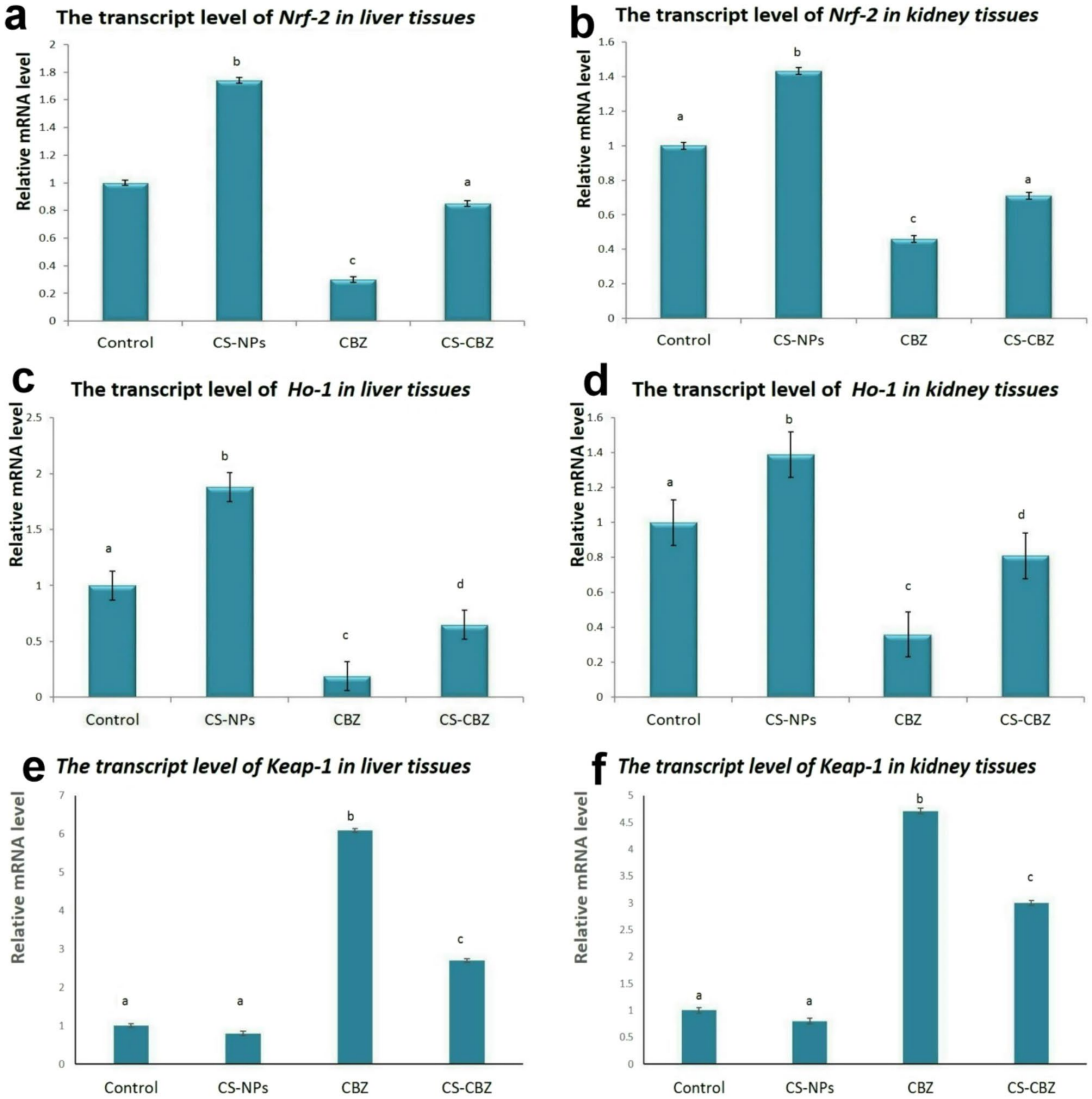
Bar charts representing; (**a**) Nrf2 transcript level in liver, (**b**) Nrf2 transcript level in kidneys, (**c**) HO-1 transcript level in liver, (**d**) HO-1 transcript level in kidneys, (**e**) Keap-1 transcript level in liver, (**f**) Keap-1 transcript level in kidneys. Values represented as Mean ± SD. Values with different letters means significant difference at *P* ≤ 0.05.

The group receiving CBZ showed significant downregulation of the transcript levels of Nrf2 and HO-1 genes with upregulation of Keap1 gene in both liver and kidneys compared with the control group. Co-administration of CS-NPs with CBZ abled to upregulate the protective genes (Nrf2 and HO-1) and downregulate Keap1 gene compared with CBZ receiving group (Fig. 6).

## Discussion

Carbendazim is the most widely used systemic fungicide in agricultural and veterinary practices^30^. It is also considered an environmental pollutant as it leaves residues in crops, food chains, soil, and water causing severe dangerous effects in different body organs^31^. Recently, nanotechnology has been used in several agricultural purposes as nano-agrochemicals, nano-fertilizers, or as encapsulating materials^32^. Chitosan nanoparticles (CS-NPs) is extensively used in agricultural field as growth promotor to increase the shelf life of some plants and crops^33^. CS-NPs caught the attention of many researchers and found many uses due to its antioxidant, non-toxic and biocompatible nature^34^. Despite the many uses of CS-NPs, its antioxidant mechanism hasn’t been studied yet. For this reason, the current study aimed to explore the mechanistic way of CS-NPs to minimize the hepatorenal toxicity induced by CBZ in rats. In addition, new formula of CS-NPs was prepared by using ascorbic acid solution instead of acetic acid to increase its antioxidant effect even if administered at very low dosage level.

The results of the present study showed a significant increase in MDA levels and a decrease in TAC, GSH, and catalase activity in the group receiving CBZ suggesting oxidative stress damage on both liver and kidneys. These findings together with the down-regulation of Nrf2 and Ho1 genes, and upregulation of Keap1 gene confirmed that the main mechanism of CBZ-induced hepatorenal toxicity is oxidative stress. Oxidative stress is defined as the imbalance between oxidants and antioxidants that lead to the disruption of redox signals causing molecular damage^35^. Malonaldehyde (MDA) is the end product of lipid peroxidation and its increase supports the hypothesis of oxidative stress involvement^36,37^. While TAC measures the total antioxidant components and its high level indicates a high response against free radicals^38^. On the other side, catalase is an antioxidant enzyme catalyzing the decomposition of H_2_O_2_^39^, whereas the reduced glutathione (GSH) is required for several cell processes associated with abnormal changes in the maintenance and regulation of the thiol-redox status^40^. It exerts a potent antioxidant role through direct interaction with reactive oxygen species (ROS) or by operating as a cofactor for the peroxidase enzyme^41^. CBZ can induce the excessive generation of reactive oxygen and nitrogen species by altering the oxidants/antioxidants balance by promoting lipid peroxidation (LPO) and depleting both enzymatic and non-enzymatic cellular antioxidants leading to a condition of oxidative stress and cell death^42,43^. ROS overproduction initiates the processes of DNA and cell membrane damage via lipid peroxidation and protein degradation^44^.

CBZ induced oxidative stress and can disturb various parts of cellular signaling and homeostasis through mediating redox signaling and depletion of antioxidant reservoirs^45^. Exogenous stimuli such as inflammatory cytokines increase intracellular ROS that contributes to the severity of pathological disorders^46^. One of the main proteins that control the redox signaling in mitochondrial oxidative phosphorylation is cytochrome c (Cc), the functions of which are regulated by the phosphorylation of several residues^47^. Under oxidative stress conditions, Cc acts as a programmed cell death inducer^48^. During early apoptosis, Cc population tightly bound to the mitochondrial membrane triggers the peroxidation of phospholipids^49^.

From the molecular aspect, the nuclear factor erythroid 2-related factor 2 (Nrf2) is a transcription factor play a pivotal role in the regulation of cellular redox balance and activates the natural antioxidants in mammals^50^. Down-regulation of Nrf2 could reduce the antioxidant defense, such as superoxide dismutase (SOD), glutathione peroxidase (GSH-Px) and heme oxygenase-1 (HO-1), and glutathione reductase^51^. On the other hand, Kelch-like ECH-associated protein1 (Keap1) is a master negative regulator of Nrf2 and its up-regulation reflects the oxidative stress damage^52^. The main intracellular sensor that monitors ROS is Keap1 gene^53^. The current study suggests that CBZ induced hepatorenal toxicity through prolonged ROS generation that activates Keap1 and results in its ability to degrade Nrf2 and down-regulates the expression of antioxidant factors as HO-1^54,55^.

Our results reported the highest values of both liver and kidney biomarkers in CBZ receiving group suggesting hepatocellular and renal damage that reflects on the microscopic pictures and showed severe cellular injury and interstitial inflammation in both liver and kidney sections. These findings may be related to the oxidative stress induced by CBZ via continuous ROS production that leads to cellular damage via necrosis or apoptosis^56^. Apoptosis is another mechanism of CBZ-induced hepatorenal toxicity in the current study and may be mediated by both ROS and nitric oxide (NO) production^57^. ROS overproduction causes mitochondrial membrane permeability via opening the transition pores leading to the release of cytochrome-c and activating several caspase cascades^58,59^. Among the effector caspases, Casp-3 is responsible for initiating the hallmarks of the degradation phase of apoptosis, including DNA fragmentation, cell shrinkage, and membrane blebbing and has a distinct role in intrinsic apoptosis^60^. Moreover, NO is an intercellular messenger that has been recognized as one of the most versatile players in the immune system and inflammatory process^61^. The inducible nitric oxide (iNOS) is most commonly associated with inflammatory conditions in which NO is produced in large amounts^62^.

Our results showed that co-administration of CS-NPs with CBZ could reduce all the studied toxicological parameters affirm to prevent both liver and kidney organs from being damage. The novel formulation of CS-NPs exerts a potent antioxidant effect manifested by a significant decrease in MDA value and an increase in TAC, GSH, and catalase activity. The results of the present study suggest that CS-NPs had the ability to protect the hepatic and renal cell membrane from oxidative damage through down-regulation of Keap1 that activates Nrf2/HO1 signaling pathway^63^. The Nrf2/HO-1 system activates cascade of events which in the end, affects oxidative status of the cells and provides robust protection against oxidative challenge^64^. Under the influence of CBZ-induced oxidative stress, CS-NPs had the ability to induce several antioxidants and anti-apoptotic proteins, including HO-1 via activation of Nrf2. Since Nrf2 is ubiquitously expressed, it plays a critical role in protecting many cell types and organ systems from oxidative stress and an array of toxic insults^65^. Previous studies reported that administration of rats with 200 mg/kg Chitosan had the ability to reduce the liver thiobarbituric acid reactive substances (TBARS) and elevate the antioxidant enzyme activities including catalase and superoxide dismutase^66^. Another study revealed that CS-NPs administration at a dosage level of 280 mg/kg demonstrated considerable improvements in rat’s liver biomarkers, oxidant/antioxidant balance, and the microscopic picture^67^.

CS-NPs can also improve the microscopic picture of both liver and kidney sections and restore liver and kidney biomarkers to the normal levels. In addition, our results revealed for the first time that CS-NPs have strong anti-apoptotic and anti-inflammatory effects manifested by weak immunohistochemical staining of casp-3 and iNOS in both liver and kidney sections. The advantage of this novel CS-NPs formula over other CS-NPs is that novel CS-NPs formula has greater antioxidant effects which could be related to both chitosan and ascorbic acid. The antioxidant capability of CS-NPs is supposed to operate by means of a direct radical scavenging mechanism^68^ such as super oxide radical and hydroxyl radical^69^ or indirectly, via metal chelation, which could block the ROS generation and lipid oxidation^70^. CS-NPs are known to increase the protein expression of Nrf2 and the mRNA levels of NQO1, HO-1^71^. Notably, activation of the Nrf2 pathways has been demonstrated to be involved in suppressing inflammatory responses^72^. It is reported that chitosan could induce the innate immune cells to release a wide range of pro-and anti-inflammatory cytokines, chemokines, growth factors and bioactive lipids^73^. The new formula using ascorbic acid has increased the antioxidant activity by suppressing free radical generation and attenuating oxidative damage^74^. It is well known that ROS is the main cause of cell death so, decreasing ROS will decrease the incidence of cell death^75^. Ascorbic acid is considered an anti-inflammatory agent^76^, and can be attributed to its ability to modulate the DNA binding activity of nuclear factor kappa-B^77^, as it can reduce plasma levels of inflammatory mediators TNF-ὰ and IL-6^78^.

## Conclusion

From the result of the current study, we can conclude that the newly prepared CS-NPs have a potent capacity to reduce the hepatorenal toxicity induced by CBZ via the antioxidant and anti-inflammatory properties of both chitosan and ascorbic acid. Chitosan up-regulates the expression of Nrf2 and the natural antioxidant HO-1, which diminish the hepatorenal oxidative stress damage and inflammation induced by CBZ. In addition, using ascorbic acid in CS-NPs preparation had the advantage to increase its antioxidant and anti-inflammatory capacity via scavenging free radicals and modulating the expression of inflammatory proteins. Our novel CS-NPs formula has potent anti-inflammatory and anti-apoptotic effects by diminishing the iNOS and caspase-3 expression. We recommend using these newly formulated CS-NPs as an antioxidant agent for the treatment of several disorders associated with oxidative stress damage. In addition, CS-NPs may be used safely as a nanocarrier for several agrochemicals as fertilizers and pesticides, but further studies are needed to confirm the safety of these newly formulated nanoconjugates.

## Data Availability

All data generated or analyzed during this study are included in this published article.
